# A Cross-Sectional Pilot Study of Probable Sarcopenia in Hemodialysis Patients

**DOI:** 10.3390/life16040649

**Published:** 2026-04-12

**Authors:** Juan Rodríguez-Mansilla, Jaime Becerra Fernández, María Victoria Martín Hidalgo-Barquero, María Jiménez-Palomares, Blanca González-Sánchez, Elisa María Garrido-Ardila

**Affiliations:** 1 ADOLOR Research Group, Department of Medical-Surgical Therapy, Medicine Faculty and Health Sciences, University of Extremadura, 06006 Badajoz, Spain; jrodman@unex.es (J.R.-M.);; 2 ICOT Arnao Hospital, 35200 Telde, Spain; jaimebecerraf01@gmail.com; 3 Nephrology Department, Badajoz University Hospital, 06006 Badajoz, Spain; mariavictoria.martin@salud-juntaex.es

**Keywords:** sarcopenia, hemodialysis, chronic kidney disease, muscle loss, physical exercise, muscle strength

## Abstract

Chronic kidney disease is defined as a progressive pathology that affects more than 10% of the world’s population, affecting waste filtration capacity. Sarcopenia, characterized by loss of muscle mass and strength, is a common complication in patients with chronic kidney disease undergoing hemodialysis. It is associated with inflammation, malnutrition and reduced quality of life. Hemodialysis is the fundamental treatment for people with chronic kidney disease, as it is key to the elimination of toxins from the body. Objective: The objective of this study was to determine the prevalence of probable sarcopenia in patients with chronic kidney disease in the Dialysis Unit of Extremadura (Spain). Material and Methods: This is a descriptive study in which 33 patients with chronic kidney disease receiving hemodialysis were selected as participants in the assessment of functional capacity and physical fitness. The procedure was performed prior to the dialysis session. Socio-demographic, clinical and physical variables were assessed. The assessment of probable sarcopenia was carried out using manual grip strength test (dynamometry), physical performance (4-meter walk test) and phase angle (PhA) (single frequency 50 Hz bioimpedance). The Charlson Comorbidity Index (CCI) was used to determine the severity of chronic disease and its impact, and analytical variables such as albumin, C-reactive protein (CRP), Neutrophil/Lymphocyte Index (NLI), Lymphocyte–Platelet Index (LPI) and total protein (TP), among others, were also included. Results: The prevalence of probable sarcopenia was 93.9% according to the criteria for muscle strength and physical performance (EWGSOP2). PhA showed statistically significant differences between the groups with and without sarcopenia (*p* = 0.039), suggesting its usefulness as a nutritional marker. No statistically significant differences were found between sarcopenia and age, albumin, Neutrophil/Lymphocyte Index or C-reactive protein (*p* > 0.05). Conclusions: There is a high prevalence of probable sarcopenia, associated with decreased handgrip strength and gait speed in patients with chronic kidney disease in hemodialysis. In addition, PhA stands out as an influential factor in the development of sarcopenia.

## 1. Introduction

Chronic kidney disease is defined as an alteration in kidney function that lasts at least 3 months. It is a progressive pathology with high morbidity and mortality. The kidneys do not function properly, and some of their functions are impaired, such as filtering wastes and excess fluids from the blood, as well as regulating the balance of electrolytes [1]. This pathology is a progressive and irreversible condition that affects more than 10% of the world’s population (800 million people) [2].

The treatment of chronic kidney disease is based on hemodialysis. Hemodialysis is a type of dialysis, considered a general procedure used to treat chronic renal failure and end-stage renal disease [3]. It is based on the filtration of toxins and water from the blood by means of a machine, normalizing the acid–base balance to finally maintain a homeostasis in the organism of subjects undergoing this treatment [4].

Several studies confirm that the quality of life of hemodialysis patients is usually worse compared to the general population in their age group. Hemodialysis sessions, being a replacement treatment necessary to continue living, lead to a series of alterations in the daily activities of patients’ lives. All of this influences psychological, socio-familial, economic and occupational changes [3,5]. These patients experience a notable physical deconditioning, which, together with a high prevalence of frailty, generates a vicious circle of deterioration and reduced quality of life [6,7,8,9,10].

The technology used today in dialysis units requires that patients mostly move to a center with facilities adapted to their treatment conditions. These commutes result in an intermittent treatment schedule, dietary limitations and restricted mobility, along with the onset of fatigue and nausea, caused by the accumulation of uremic toxins and the rapid elimination of solutes and fluids [11]. According to the 2020 Hemodialysis Guidelines, the usual recommended time for patients undergoing this type of treatment is at least three times per week, with an average duration of 4 h [4,11].

Based on the above, chronic kidney disease favors the development of sarcopenia, which is significantly associated with patients undergoing hemodialysis treatment [12]. Sarcopenia is a pathology characterized by the gradual loss of skeletal muscle function, strength and mass exclusively, related to low physical fitness. Its etiology may be related to both ageing and other underlying factors, such as physical inactivity, malnutrition, low protein intake or chronic diseases [13,14].

According to scientific evidence, several risk factors for sarcopenia in chronic kidney disease patients on hemodialysis have been identified, including older age, female sex, lack of physical activity, albumin, pre-albumin, pre-dialysis creatinine, low Body Mass Index (BMI), malnutrition, high inflammatory status, tumor diseases and muscular dystrophy [15].

For the identification and diagnosis of sarcopenia, the consensus of the European Working Group on Sarcopenia in Older People (EWGSOP2) is currently the most widely accepted framework in Western clinical practice [13]. This model updates the definition by focusing on muscle strength as the primary indicator of suspicion, followed by confirmation through the assessment of muscle mass or quality, and the determination of severity via physical performance [16]. The application of these criteria is essential in hemodialysis patients, as it enables the early detection of muscle vulnerability in a population at high protein-energy risk.

For muscle mass, dual-energy X-ray absorptiometry (DXA) and bioimpedance analysis (BIA) are used. The measurement is performed in the areas where there is the greatest loss of body mass, i.e., the arms and knees [17], and with respect to physical performance, research supports that the best way to perform a study of this variable in by measuring walking speed [18,19].

Therefore, the assessment of patients with chronic kidney disease that includes functional tests is recommended, as these strategies have been shown to improve the detection of sarcopenia [19], allowing early interventions such as physical exercise or nutritional support for the optimization of quality of life [5,6,10].

Currently, the scientific literature evidences the benefits of physical exercise in hemodialysis patients, such as improved physical function, exercise capacity, health-related quality of life, reduced cardiovascular risks, control of comorbidities, etc. [20]. In addition, physical activity reduces, in part, episodes of hypotension and reduces levels of C-reactive protein (CRP), a key marker of inflammation [10].

Despite the established link between hemodialysis and sarcopenia, current evidence has significant methodological limitations that this study aims to address. Much of the previous research has focused on relatively young cohorts or those with strict exclusion criteria regarding comorbidity, which limits the applicability of the findings to real-world clinical practice [21]. Previous studies have noted that the systematic exclusion of patients with a high disease burden prevents an understanding of the complex interaction between multimorbidity and muscle wasting [22]. Furthermore, there is considerable heterogeneity in diagnostic protocols, thus hindering the standardization of results in dialysis units with limited resources [16]. Unlike these studies, the present study evaluates a “real-world” cohort characterized by advanced age and high multimorbidity, using accessible, low-cost screening tools such as dynamometry and phase angle, which have been shown to be effective for early detection in routine hospital settings [23].

Based on all of the above, the aim of this cross-sectional pilot study was to determine the prevalence of probable sarcopenia in patients with chronic kidney disease in the dialysis unit of the Perpetuo Socorro Hospital of Badajoz (Spain).

## 2. Materials and Methods

The present study is an observational, descriptive and cross-sectional study.

### 2.1. Characteristics of the Sample

The target population in this study consisted of individuals receiving hemodialysis treatment at the Hospital Perpetuo Socorro in Badajoz (Spain). It included adult and elderly individuals, with an age range between 48 and 91 years, reflecting a predominantly older adult population, in line with the high prevalence of chronic kidney disease. Participants were consecutively selected from patients attending regular hemodialysis sessions at the hospital center during the data collection period. The sample size was pragmatic, and no formal sample size or power calculation was performed, given the pilot nature of the study.

### 2.2. Inclusion Criteria

The inclusion criteria included chronic kidney disease patients on hemodialysis treatment, over 18 years of age, with preserved standing and walking ability.

### 2.3. Exclusion Criteria

Patients presenting with an acute inflammatory process, severe cognitive impairment that prevented understanding the instructions, or a total inability to stand or walk independently were excluded. Furthermore, patients with unstable clinical conditions or chronic diseases (such as active malignancies or acute neurological sequels from stroke) that were the primary and sole cause of functional limitation at the time of the study were also excluded to minimize potential confounding factors. However, other stable comorbidities were recorded and analyzed through the Charlson Comorbidity Index to account for the overall disease burden of the sample.

### 2.4. Ethical Aspects

This study was conducted in accordance with the ethical principles established in the Declaration of Helsinki and with the approval of the Bioethics and Biosafety Commission of the University of Extremadura, reference number 132/2023.

All participants were informed in detail about the objectives, procedures, benefits and possible risks of the study, as well as their right to withdraw at any time without repercussions on their medical care. Prior to inclusion, each participant recorded written informed consent, guaranteeing that their participation was voluntary and fully conscious.

### 2.5. Variables of Study

Prior to the data collection, a literature review was carried out in PubMed, Science Direct and Scopus databases, among the most relevant, in addition to a manual search for information.

Sociodemographic, clinical and analytical variables included age, sex, vascular access (central line catheter or fistula), cause of chronic kidney disease, time on hemodialysis, presence of diabetes or not and the Charlson Comorbidity Index (CCI). In addition, analytical variables such as albumin, CRP, Neutrophil/Lymphocyte Index (NLI), Lymphocyte–Platelet Index (LPI) and total protein (TP), among others, were included.

### 2.6. The Assessment Tools Used Were

Dynamometry: Dynamometry was used to assess muscle groups of both upper limbs. This test has shown high sensitivity and specificity for early detection of variations in muscle mass status and improved interventions for hemodialysis patients [24,25].

This test measures the strength of patients when performing a manual grip. To perform the measurement, a hand-held dynamometer (FINGERSK, model SK101GY) was used. The users were in a standing position with both arms stretched out. The arm with which the force was measured was completely stretched and in neutral pronosupination. The order given to the patient was to perform the maximum possible force for 5 s. Two repetitions for each arm were completed, noting, as a result, the maximum value recorded [25].

Four-meter walk test: This test provides data on acceleration, walking speed and deceleration. A digital stopwatch was used to measure the time. To perform the test, a chair, a tape measure and a stopwatch were used. A distance of 4 meters was measured between the chair and a mark on the floor. The patient sat on the chair and on our signal got up from the chair and started walking at his or her usual walking pace until he or she reached the marker. For better understanding, we gave the command, “walk as if you were going down the street to buy bread”. Two attempts were made and the best time was chosen. The participant wore their usual footwear and could use their walking stick, walker or companion if required. The stopwatch was stopped once both feet had passed the 4-meter marker. A walking speed of less than 0.8 m/s was an indicator of poor physical performance [13,26].

Phase angle (PhA) assessment: PhA was measured using a 50 Hz monofrequency bioimpedance analyzer (BIA 101, Akern, Florence, Italy). This non-invasive procedure was used to assess nutritional status and cellular integrity. To ensure standardization, all patients were placed in the supine position with legs apart and arms not in contact with the torso. The measurements were performed immediately before the mid-week hemodialysis session to minimize the impact of acute changes in body fluid volume [27].

Charlson Comorbidity Index: The Charlson Comorbidity Index is a valid and reliable comorbidity index that is used in the renal population. The index contains 19 comorbid conditions, such as myocardial infarction, dementia, diabetes or hemiplegia. It is scored from 1 to 6, with the total score being the burden of comorbidity. A score of 0 indicates no significant comorbidities, while a score ≥3 indicates severe comorbidity, with increased risk of mortality [28]. To calculate the Charlson Comorbidity Index, the patient’s medical history was reviewed to identify relevant medical conditions.

Analytical parameters: Urine and blood tests are a valuable tool to assess effective intake and adherence to prescriptions by performing a biochemical assessment [29]. The following were mainly evaluated:

Serum albumin: Serum albumin is the main biochemical indicator to assess the risk of malnutrition [30,31], and is considered a key parameter in nutritional assessment [32,33]. Serum albumin levels decrease with age by approximately 0.1 g/L per year. However, age in itself is not a cause of definite hypoalbuminemia [33]. Normal albumin values are between 3.5 and 5.0 g/dL [34], and during hemodialysis treatment, patients show a reduction in this concentration, being an indicator of mortality. Low serum albumin levels may be related to alterations in hydration status in hemodialysis patients, and scientific evidence shows an association between albumin levels and inflammation [30,35].

Lymphocytes: Hemodialysis patients often present with chronic inflammation. Such inflammation is sometimes related to T-lymphocyte dysfunction, with lymphopenia and/or activation of pro-inflammatory cytokine production. Lymphocyte counts can be related to neutrophils, due to their role in the body’s immune system, resulting in the Neutrophil/Lymphocyte Index (NLI). This index is one of the most sensitive and specific biomarkers in hemodialysis patients [36,37].

CRP: CRP is an indicator of the inflammatory process in chronic kidney disease, reflecting the production of pro-inflammatory cytokines such as tumor necrosis factor-a (TNFα) and interleukin (IL-6) [38]. Monthly monitoring of CRP can help control the presence of contamination in the dialysis water, control vascular access status and ensure the proper optimization of dialysis protocols and doses [36]. According to the scientific literature, high CRP levels are associated with low serum albumin levels in patients with this condition. These data provide information about the patient’s condition, which may include a chronic inflammatory state, risk of malnutrition, increased risk of cardiovascular events and mortality [39].

Sarcopenia was screened and classified according to the EWGSOP2’s updated clinical algorithm. In this study, the presence of probable sarcopenia was identified based on low muscle strength (handgrip strength < 27 kg for men and <16 kg for women). Due to the clinical pilot nature of the study and the search for low-cost screening tools, muscle mass quantification was not performed, which is recognized as a study limitation.

### 2.7. Procedure

Data were collected at the hemodialysis unit of the Perpetuo Socorro Hospital in the city of Badajoz (Extremadura, Spain) during the months of December 2024–April 2025. The unit was visited several times, and after explaining the study procedure, 40 participants signed the informed-consent form and agreed to participate.

A personalized interview was conducted with each of them, where socio-demographic data were collected and the aforementioned tests were administered before the patients started their hemodialysis treatment. Subsequently, the data were collected and analyzed.

### 2.8. Data Analysis

The data collected were analyzed to describe the socio-demographic, clinical, functional and biochemical characteristics of hemodialysis patients, as well as to explore the relationship between probable sarcopenia and biopsychosocial variables. Statistical analysis was performed with the Jamovi 2.6.44 statistical software.

Continuous variables, such as age, hemodialysis time, CCI, handgrip strength (dynamometry), gait speed (4 m walk test), creatinine, albumin, PT, hematocrit, INL, ILP and CRP, were described by measures of central tendency (mean and median) and dispersion (standard deviation, minimum and maximum range). The distribution of these variables was assessed visually by histograms to determine normality.

Categorical variables, including sex, type of vascular access (catheter or fistula), cause of chronic kidney disease and presence of diabetes, were expressed as absolute frequencies and relative percentages. Results were graphically represented by bar charts and frequency tables.

To compare variables between groups with and without probable sarcopenia (defined by low handgrip strength [<27 kg for men, <16 kg for women] and/or low gait speed [≤0.8 m/s]), Student’s *t*-test was used for normally distributed variables, and the Mann–Whitney U test for non-normal variables. Variables analyzed included age, PhA, albumin, INL and CRP.

Pearson correlation coefficients were calculated to assess the relationship between probable sarcopenia and continuous variables such as PhA, albumin, INL and CRP, with a significance level set at *p* < 0.05.

## 3. Results

**Figure 1:**
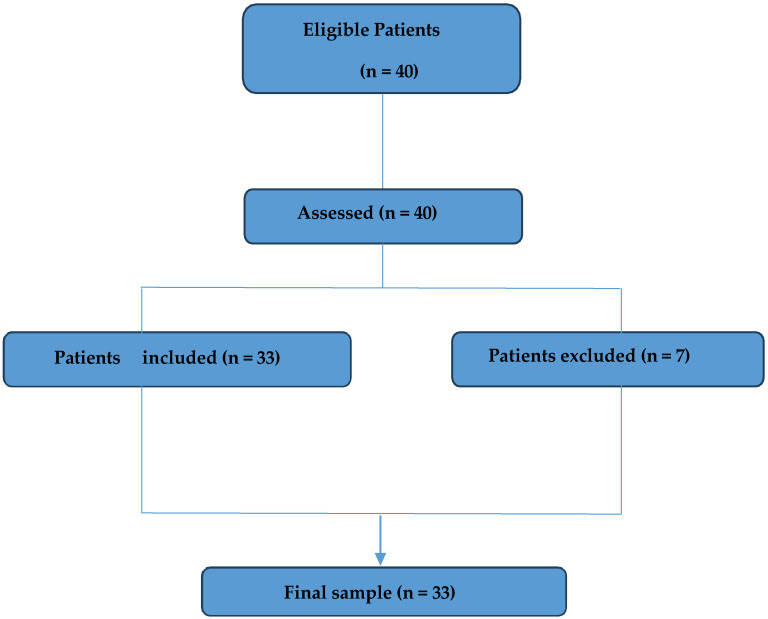
Flowchart of the study.

As indicated above, of the 40 participants initially recruited for the study, seven of them were excluded because they were not able to stand or walk independently. The final sample consisted of 33 participants (Figure 1).

The data for the variables age, time on dialysis and Charlson Index are summarized in Table 1. The mean age was 75.45 years, with a median of 78.00 years [SD = 9.36] [48.0–88.0]. Time on dialysis showed a mean of 8.92 years and a median of 6.00 years [SD = 7.75] [0.30–28.00]. The Charlson Index had a mean of 8.27 and a median 8.00 [SD = 2.05] [3,4,5,6,7,8,9,10,11,12] (Table 1). Further information can be found in the following table and graphs.

**Figure 2:**
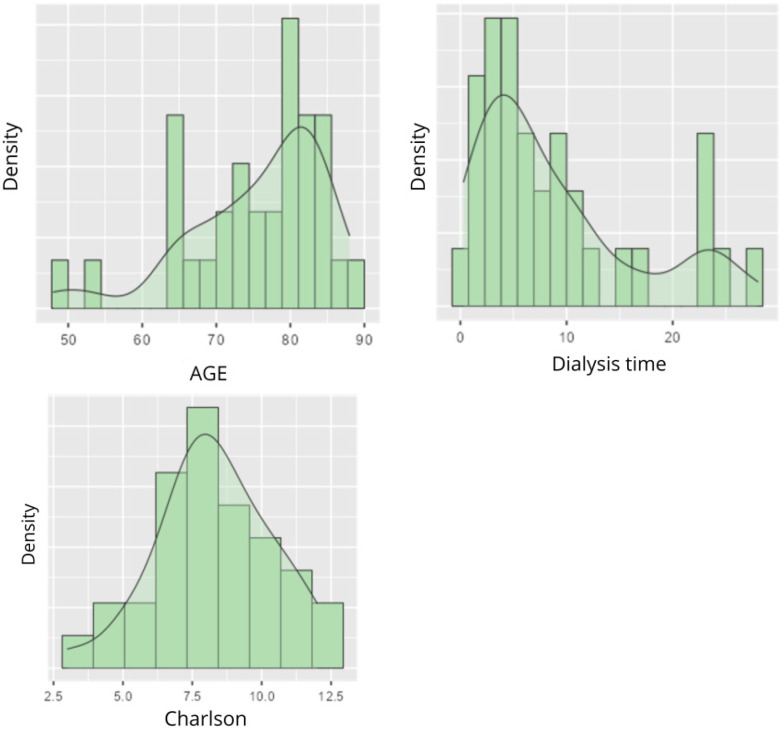
Variables: Age, time on dialysis and Charlson Comorbidity Index.

The age distribution showed a higher density between 75 and 85 years, indicating a majority of older patients (Figure 2). The variable time on dialysis showed an asymmetric distribution, with a main peak between 0 and 5 years, and more dispersed values. Short times predominate, although some patients have been on treatment for longer periods, contributing to the development of comorbidities and progression of chronic kidney disease (Figure 2). The Charlson comorbidity Index was more evenly distributed, with a peak around 6.0–10.0, indicating that the population studied has a significant burden of comorbidities (Figure 2). This suggests the need to involve personalized approaches in those patients with a higher burden of comorbidity and further progression of pathology.

**Figure 3:**
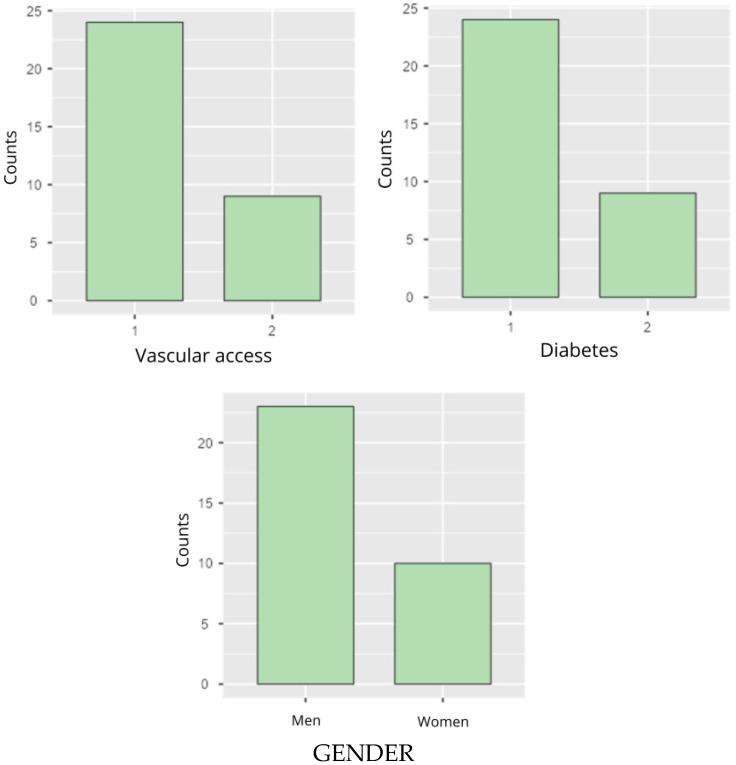
Variables: Vascular access, diabetes, and gender.

The variable vascular access was distributed in 72.7% of participants (*n* = 24) with vascular access via catheter, while 27.3% (*n* = 9) had vascular access via fistula (Figure 3 and Table 2). Regarding the cause of disease, unfiltered chronic kidney disease was the most frequent, with 30.3% (*n* = 10), followed by diabetic nephropathy, with 24.2% (*n* = 8), and other causes, such as hereditary renal disease and glomerulonephritis with, 9.1% and 15.2% (*n* = 3 and *n* = 5, respectively) (Table 2). For the variable diabetes, 72.7% (*n* = 24) of the participants were classified in category 1, meaning that they have diabetes, and 27.3% (*n* = 9) were in category 2, meaning that they do not have diabetes (Figure 3 and Table 2). In relation to gender distribution, 69.7% of the participants (*n* = 23) were male, while 30.3% (*n* = 10) were female (Figure 3 and Table 2). The following table and graphs discuss the variables in more detail.

### 3.1. Analytical Variables

**Figure 4:**
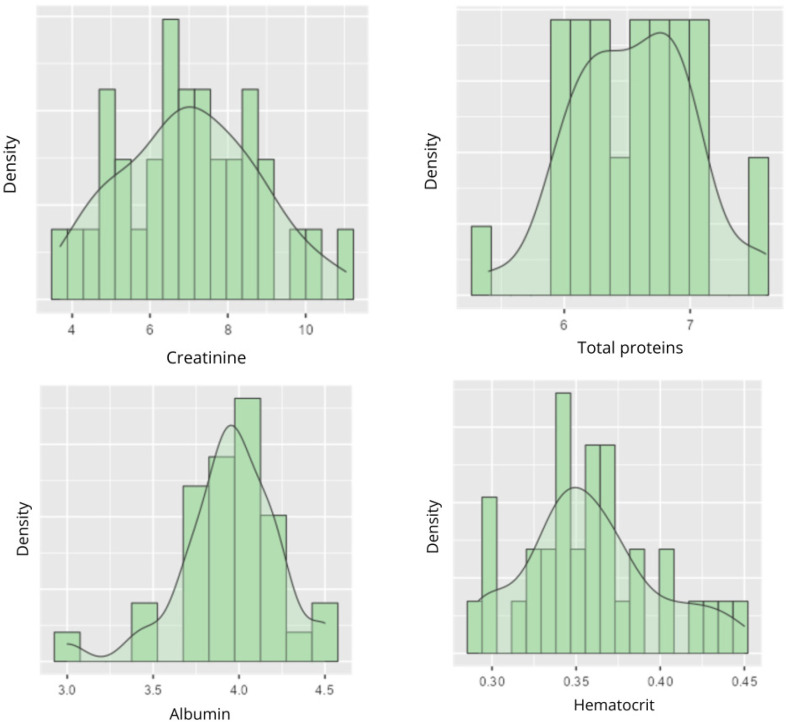
Variables: Creatinine, total protein, albumin and hematocrit.

**Figure 5:**
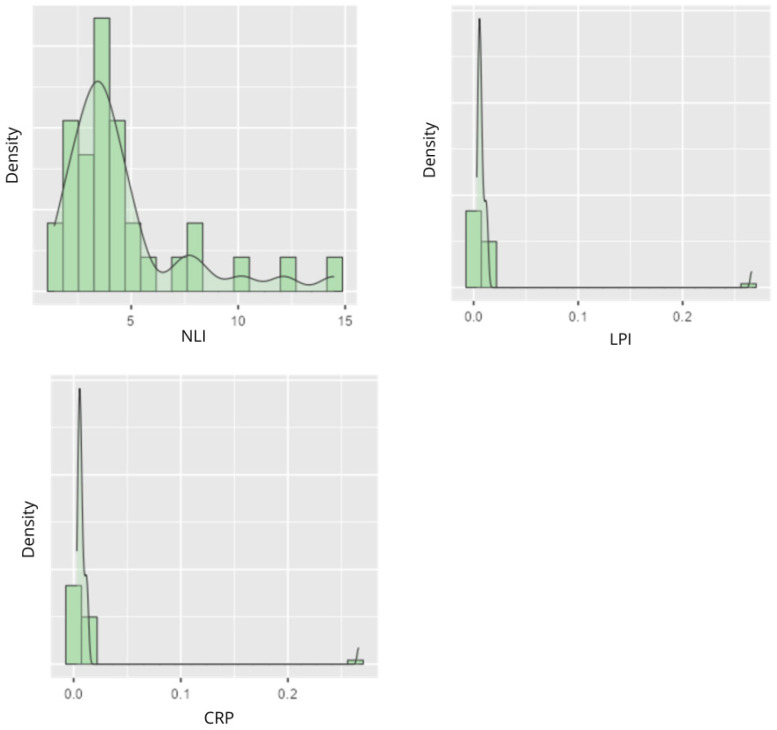
Variables: Neutrophil/Lymphocyte Index (NLI), Lymphocyte–Platelet Index (LPI) and C-reactive protein (CRP).

The clinical variables observed in the analysis are detailed in Table 3. Creatinine showed a mean of 7.0318 mg/dL, with a median of 6.87000 mg/dL [SD = 1.728] and [3.6900–11.040] (Figure 4). Total protein had a mean of 6.5455 g/dL, with a median of 6.60000 g/dL [SD = 0.477] and [5.40000–7.600] (Figure 4). Albumin showed a mean of 3.9455 g/dL, with a median of 4.00000 g/dL [SD = 0.298] and [3.00000–4.500] (Figure 4). Hematocrit showed a mean of 0.3591, with a median of 0.35800 [SD = 0.040] and [0.29200–0.450] (Figure 4). INL had a mean of 4.6100, with a median of 3.72000 [SD = 2.971] and [1.41000–14.470] (Figure 5). Lymphocyte–Platelet Index presented a mean of 0.0150, with a median of 0.00633 [SD = 0.045] and [0.00300 to 0.266] (Figure 5). C-reactive protein showed a mean of 9.1636 mg/L, with a median of 5.00000 mg/L [SD = 10.62] and [0.30000–49.800] (Figure 5).

The distribution of creatinine showed a peak around 6–8 mg/dL, with a slight asymmetry towards higher values (Figure 4). Total proteins were concentrated between 6 and 7 g/dL, with a lower density at the extreme values (Figure 4), while albumin presented a peak around 4.0 g/dL. There is a proportion of individuals with levels close to the inner limit of normality (3.5 g/dL), indicative of possible conditions of malnutrition and/or chronic inflammation (Figure 4). Hematocrit showed a pronounced peak around 0.40 with moderate scatter (Figure 4), and Neutrophil/Lymphocyte Index had a prominent peak around 5, which may relate to inflammatory or infectious conditions in the case of elevated values (Figure 5). Lymphocyte–Platelet Index and C-reactive protein showed distributions with peaks at low values (0.0–0.1 and 0.0–10.0 mg/L, 34 respectively). As for C-reactive protein, a second peak is observed with values between 20 and 30. From 30, the density progressively decreases (Figure 5).

**Figure 6:**
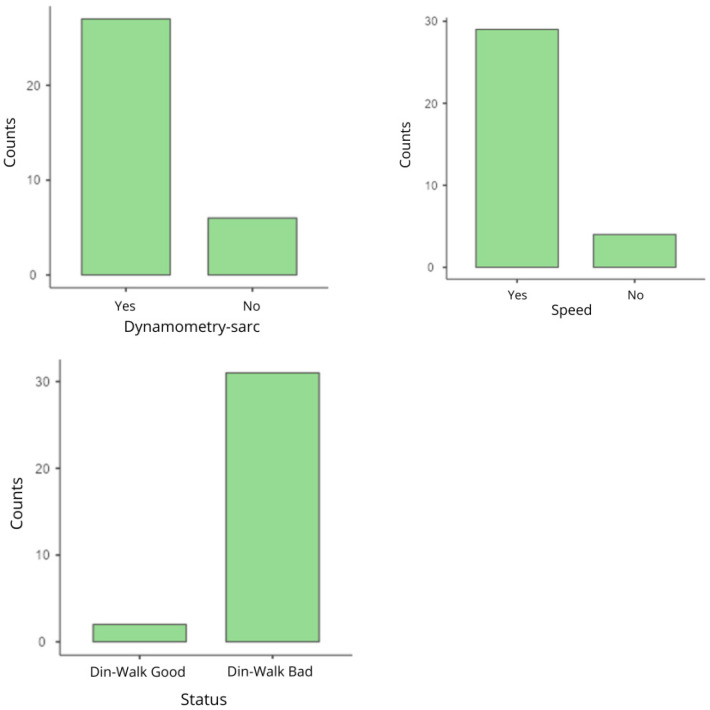
Variables: Dynamometry–sarcopenia, speed–sarcopenia and Sarcopenia.

The data of the variables sarcopenia–dynamometry, sarcopenia–speed and sarcopenia are summarized in Table 4. Regarding sarcopenia–dynamometry, 81.8% of the participants (*n* = 27) were classified as “yes” to take into account the possibility of developing sarcopenia, while 18.2% (*n* = 6) were classified as “no” (Figure 6). Regarding speed-sarcopenia it was observed that 87.9% of cases (*n* = 29) were classified as “yes”, while 12.1% of cases (*n* = 4) were classified as “no” (Figure 6). As for the variable sarcopenia, it was divided into two groups of patients, those with the dynamometry test and the 4 m walk test doing well, and patients with both tests doing poorly. In total, 6.1% (*n* = 2) were identified as “dyn–gait good”, with no probability of developing probable sarcopenia, while the remaining 93.9% (*n* = 31) were identified as “dyn–gait bad” (Figure 6).

The following histograms illustrate these distributions, highlighting the prevalence of “yes” in the variables dynamometry–sarcopenia and speed–sarcopenia, and the majority of “dynamometry–sarc bad” in the variable sarcopenia.

### 3.2. Comparative Analysis

To evaluate the differences between the groups according to the sarcopenia variable, Student’s *t*-test and the Mann–Whitney U test were used, applied to the variables PhA, age, albumin, Neutrophil/Lymphocyte Index and C-reactive protein. The following table (Table 5) shows the data collected with the aforementioned statistical analysis.

The results of the variables analyzed in Table 5 show no significant differences (*p* > 0.05) in the variables age, albumin, Neutrophil/Lymphocyte Index and C-reactive protein. On the other hand, the PhA variable shows significant differences between the groups with and without probable sarcopenia. This indicates that the PhA may be an influential factor in the development of probable sarcopenia.

### 3.3. Correlation Analysis

Table 6 presents the results obtained from the Spearman correlation matrix, carried out to explore the possible monotonic relationships among the selected variables: PhA, albumin, Neutrophil/Lymphocyte Index and C-reactive protein. For each pair of variables, the Spearman correlation coefficient (R), degrees of freedom (gl) and corresponding *p*-value were calculated to determine the statistical relevance of the associations. The results indicate that, in general, the variables do not show statistically significant monotonic correlations at the conventional level of *p* < 0.05. The relationship between PhA and albumin stands out with a moderate positive correlation (R = 0.522) and a high level of statistical significance (*p* < 0.001), suggesting that higher albumin levels tend to be associated with better cellular integrity reflected by the PhA. In contrast, the correlations of PhA with NLI (R = –0.196; *p* = 0.231) and with CRP (R = –0.213; *p* = 0.193) are weak and not statistically significant, indicating that no clear monotonic trend can be inferred between these inflammatory markers and the PhA in this sample. Similarly, the correlation between albumin and NLI (R = 0.036; *p* = 0.830) and between albumin and CRP (R = –0.243; *p* = 0.136) remains weak and lacks statistical significance. The only inflammatory marker relationship showing statistical significance is the one observed between NLI and CRP (R = 0.358; *p* = 0.025), representing a modest but statistically meaningful monotonic association. This suggests that increases in systemic inflammation reflected by CRP tend to accompany increases in NLI, a relationship consistent with their shared inflammatory nature. Although most correlations do not reach statistical significance, the observed trends may gain clarity with a larger sample size, particularly those that approach significance thresholds. These findings contribute to our understanding of the interactions between nutritional, cellular and inflammatory parameters in the studied population.

## 4. Discussion

It is essential to note that, according to the EWGSOP2 consensus, muscle strength is currently the most reliable measure of muscle function and the primary predictor of adverse outcomes [13]. In hemodialysis patients, probable sarcopenia (detected by dynamometry) is a sufficient clinical marker to initiate nutritional and exercise interventions, as loss of strength precedes loss of muscle mass and is more debilitating than loss of muscle mass in this population [40,41].

The aim of the study was to determine the prevalence of probable sarcopenia in patients with chronic kidney disease in the Dialysis Unit of the Hospital Perpetuo Socorro in Badajoz, Spain. This research has shown a high prevalence of probable sarcopenia in chronic kidney disease patients on hemodialysis treatment in this sample, with a percentage of 93.9% according to the criteria for muscle strength and physical performance established by EWGSOP2 [13]. This finding suggests a relationship between advanced chronic kidney disease and probable sarcopenia. According to Duarte et al. [42], the prevalence of sarcopenia in people with chronic kidney disease is high due to pathophysiological factors such as chronic inflammatory state, metabolic acidosis, insulin resistance or protein hypercatabolism [12,42,43]. The exceptionally high prevalence of probable sarcopenia observed in the sample of our study (93.9%) contrasts with the lower rates (ranging from 30% to 70%) reported in the previous literature. This discrepancy represents a key finding of our research: while existing studies often apply strict exclusion criteria that filter out the most fragile patients, our pilot study reflects the “real-world” clinical profile of modern dialysis units. The advanced age of our sample (mean 75.45 years) and the high comorbidity burden (mean CCI 8.27) suggest that in the hemodialysis population, probable sarcopenia is not just a common complication but a condition that could easily be associated with chronic kidney disease, and therefore, these patients could benefit from systematic screening through accessible tools like those evaluated here.

Statistically, the PhA showed significant differences (*p* = 0.039), thus supporting its clinical utility as a nutritional marker and predictor of muscle mass loss. These findings represent associations within a cross-sectional design. Kosoku et al. [44] found that a PhA ≤ 4.46° is associated with an increased risk of sarcopenia in renal patients [44]. Although in this analysis, variables such as albumin, Neutrophil/Lymphocyte Index or C-reactive protein have not reached a remarkable statistical significance, previous studies have pointed out their importance as indicators of inflammation and malnutrition, considering their relationship with the evolution of sarcopenia [45,46]. In this study, the results showed a significant increase in the risk of probable sarcopenia in the renal patients included.

There was no statistically significant relationship between age and probable sarcopenia, due to a low age variability in the sample (mean of 75.45 years). Scientific evidence supports the fact that, in geriatric populations with chronic kidney disease, age is no longer a differentiating predictor, with inflammatory status, malnutrition, the presence of comorbidities and physical inactivity being of greater prognostic relevance [47,48]. Recent studies, such as that of Sabatino et al. (2021) [43], reinforce the idea of implementing effective and personalized preventive strategies at older ages, as doing so can attenuate the loss of muscle mass and preserve functional capacity.

On the other hand, no significant differences were found in the Neutrophil/Lymphocyte Index and C-reactive protein variables. Yuan et al. (2023) [45] have determined that both Neutrophil/Lymphocyte Index and C-reactive protein are related to the inflammatory processes that take place in this type of patient, leading to muscle loss, the appearance of frailty and a poorer quality of life. With these results, future studies with a larger number of participants are recommended, as they would help to determine a statistically significant relationship between both variables and sarcopenia. In addition, this could help clarify the evidence to support nutritional and anti-inflammatory treatments to manage the evolution of sarcopenia considering the influence of the Neutrophil/Lymphocyte Index and C-reactive protein.

The high mean Charlson Index (8.27) indicates a high burden of comorbidities among patients in this study, which supports the need for a comprehensive and interdisciplinary therapeutic approach. The scientific literature supports the need for such strategies, including individualized nutritional, physical and psychological intervention [46,49]. In addition, this approach improves adherence to treatment, with increased autonomy; individualized care; and optimized clinical, functional and psychosocial outcomes.

Although the impact of physical exercise was not directly assessed in this study, scientific evidence supports the fact that physical exercise is considered one of the fundamental therapeutic approaches for the management of sarcopenia in dialysis units. According to Mori et al. [12] and Sabatino et al. [43], intradialytic physical exercise helps to preserve skeletal muscle, improve functional capacity and decrease the protein catabolism common in this patient profile. In addition, it can prevent functional deterioration, reduce fatigue and improve quality of life [20]. The initiation of this type of therapeutic approach influences the decrease in inflammatory markers such as CRP, although no statistically significant differences were found in this study. Manfredini et al. [10] and Cruz-Jentoft et al. [13] established that, by reducing levels of this variable, an improvement in both muscle mass and function can be achieved. Therefore, the results obtained in the present study reinforce the need for individualized physical exercise programs supervised by physiotherapists, whose presence in multidisciplinary dialysis teams is key. The intervention can be carried out during hemodialysis sessions, where patients remain at rest for several hours.

### Limitations and Proposals for Improvement

Despite the relevant findings, this study has several limitations that should be acknowledged. The main limitation is the lack of direct muscle mass measurement, which prevented a definitive diagnosis of sarcopenia according to the full EWGSOP2 algorithm. However, identifying probable sarcopenia through handgrip strength remains a powerful and accessible tool for early clinical screening in dialysis units, where complex body composition sessions are not always feasible.

Although direct muscle mass quantification was not performed in the present study, PhA was obtained through bioelectrical impedance analysis. This parameter is associated with both the quantity and quality of muscle mass and has been proposed as a potential indicator of cellular functional status and membrane integrity. The diagnosis of suspected sarcopenia is established, according to the EWGSOP2 consensus, through the assessment of muscle strength. Confirmation of the diagnosis requires demonstrating reduced muscle mass by dual-energy X-ray absorptiometry (DEXA), computed tomography, magnetic resonance imaging or bioelectrical impedance analysis. Severity is subsequently determined through the evaluation of muscle power. However, recent evidence indicates that muscle strength shows a stronger correlation with the presence and progression of sarcopenia than the quantification of muscle mass alone. Several studies have demonstrated that the loss of muscle strength predicts mortality, frailty and hospitalization more reliably than muscle mass. This observation has led to a conceptual shift in which strength has emerged as the principal functional and clinical predictor, whereas muscle mass is considered a complementary parameter. Consequently, the diagnostic process becomes simpler by relying on strength- and power-based tests that are easy to apply in routine clinical practice, facilitating the screening and early identification of probable sarcopenia. In this context, the measurement of muscle mass—requiring specialized equipment not available in many healthcare centers—may not be strictly essential for the initial detection of the disease [40,50,51,52].

Additionally, the sample size and the cross-sectional nature of this study limit our ability to establish causal relationships. The cross-sectional design does not allow for causal inference between probable sarcopenia and the examined clinical or biochemical variables. Firstly, the statistical power of the research is low due to the small sample size. Therefore, the sample size could be considered a limitation of this study. However, studies conducted in hemodialysis populations tend to have small sample sizes due to several factors intrinsic to the clinical setting itself. This is a highly specific and limited population, which restricts the number of eligible patients during the inclusion period. This situation is common and has been previously described in pilot and observational studies in nephrology, where the number of patients recruited is necessarily low due to the actual availability of cases undergoing renal replacement therapy [53]. The final number of participants in our study reflects the actual availability of hemodialysis patients at the center during the defined period, as well as the need to maintain strict inclusion criteria to ensure clinical homogeneity. This justification is consistent with previous hemodialysis studies and current methodological recommendations for pilot studies [53,54,55]. Therefore, we recommend larger sample sizes in future studies to strengthen statistical validity. The cross-sectional design of this study has made it difficult to establish causal relationships of the development and progress of probable sarcopenia over time, and the effect of the interventions on patients undergoing treatment. In addition, the possible influence of specific comorbidities, such as active malignant neoplasms or neurological disorders (e.g., strokes), must be recognized as confounding factors in the development of probable sarcopenia. Although these conditions may independently promote muscle loss, our study aimed to reflect the clinical reality of an elderly population on hemodialysis (mean age, 75.45 years), in which multimorbidity is the norm rather than the exception. To mitigate this bias, we used the Charlson Comorbidity Index to quantify and adjust for overall disease burden. However, future studies with larger cohorts should consider stratified analyses to isolate the specific impact of these comorbidities on muscle mass and physical function.

It would be desirable in future research to increase the sample size to increase statistical significance, as well as to establish a longitudinal study to observe the evolution of sarcopenia and the impact of the interventions over time. In addition, it is recommended to establish intradialytic and extradialytic physical exercise programs adapted to the needs of the patients in order to improve adherence to treatment.

Based on the results of this study, we can conclude that there is a high prevalence of probable sarcopenia in patients in the dialysis unit of the Perpetuo Socorro Hospital in Badajoz (Extremadura, Spain).

In reference to functional capacity, decreased or reduced handgrip strength and gait speed are associated with the development of probable sarcopenia and poor physical performance. However, the single-center design and the predominantly elderly study population limit the external validity and generalizability of the findings to other hemodialysis settings or younger populations

The PhA, a biomarker of nutritional status, may be related as an influential factor in the development of probable sarcopenia. However, no significant association of the other analytical variables with probable sarcopenia was observed.

The results obtained in this study reinforce the importance of incorporating preventive and therapeutic strategies, such as intradialytic physical exercise supervised by physiotherapists, in order to improve muscle function, functional capacity and quality of life. However, the conclusions are preliminary and may need to be revised as larger, multicenter and longitudinal cohorts with longer follow-up become available.

## Figures and Tables

**Table 1:** Results of the variables age, time on hemodialysis and Charlson Comorbidity Index.

	N	Mean	Median	SD	Minimum	Maximum
AGE	33	75.45	78.00	9.36	48.000	88.0
TIME ON DYALISIS	33	8.92	6.00	7.75	0.300	28.0
CCI	33	8.27	8	2.05	3	12

Note: CCI, Charlson Comorbidity Index.

**Table 2:** Socio-demographic and clinical variables.

Variable	Group	Frequency	% of the Total	Accumulated %
VASCULAR ACCESS	Catheter	24	72.7%	72.7%
	Fistula	9	27.3%	100.0%
ASOCIATED ILNESSES	Non-filiated CKD	10	30.3%	30.3%
	Hereditary kidney disease	3	9.1%	39.4%
	Glomerulonephritis	5	15.2%	54.5%
	Diabetic nephropathy	8	24.2%	78.8%
	Hypertensive nephropathy	1	3.0%	84.8%
	Tubulointerstitial nephropathy	1	3.0%	84.8%
	Vascular nephropathy	3	9.1%	93.9%
	Polycystic kidney disease	1	3.0%	97.0%
	Bilateral renal tumor	1	3.0%	100.0%
DIABETES	Yes (1)	24	72.7%	72.7%
	No (2)	9	27.3%	100.0%
GENDER	Man	23	69.7%	69.7%
	Woman	10	30.3%	100.0%

Note: CKD, chronic kidney disease.

**Table 3:** Analytical variables.

	N	Loss	Mean	Median	Sd	Minimum	Maximum
CREATININE	33	0	7.0318	6.87000	1.728	3.69000	11.040
TP	33	0	6.5455	6.60000	0.477	5.40000	7.600
ALBUMIN	33	0	3.9455	4.00000	0.298	3.00000	4.500
HCT	33	0	0.3591	0.35800	0.040	0.29200	0.450
NLI	33	0	4.6100	3.72000	2.971	1.41000	14.470
LPI	33	0	0.0150	0.00633	0.045	0.00300	0.266
CRP	33	0	9.1636	5.00000	10.62	0.30000	49.800

Note: Total proteins (TP), hematocrit (HCT), Neutrophil/Lymphocyte Index (NLI), Lymphocyte–Platelet Index (LPI), C-reactive protein (CRP).

**Table 4:** Results of the physical tests.

Variable	Group	Frequency	% of the Total	Accumulated %
DYNAMOMETRY– SARC	Yes	27	81.8%	81.8%
	No	6	18.2%	100.0%
SPEED–SARC	Yes	29	87.9%	87.9%
	No	4	12.1%	100.0%
PROBABLE SARCOPENIA	Dyn–Gait good	2	6.1%	6.1%
	Dyn–Gait bad	31	93.9%	100.0%

Note: Dynamometry–sarc (dynamometry–sarcopenia), speed–sarc (speed–sarcopenia), dynamometry–gait good (dyn–gait good), dynamometry–gait bad (dyn–gait bad).

**Table 5:** Differences between the groups according to the sarcopenia variable.

		Statistics	Gl	*p*
PhA	T de Student	−2.158	31.000	0.039
	U de Mann–Whitney	17.500		0.049
AGE	T de Student	−0.070	31.000	0.945
	U de Mann–Whitney	23.000		0.570
ALBUMIN	T de Student	1.002	31.000	0.324
	U de Mann–Whitney	24.000		0.620
NLI	T de Student	−0.628	31.000	0.535
	U de Mann–Whitney	24.000		0.640
CRP	T de Student	−0.948	31.000	0.350
	U de Mann–Whitney	14.000		0.213

Note: PhA, phase angle; NLI, Neutrophil/Lymphocyte Index; CRP, C-reactive protein.

**Table 6:** Correlation matrix.

	PhA	Albumin	NLI	CRP
PhA	Spearman’s R	—	—	—
	gl	—	—	—
	*p*	—	—	—
ALBUMIN	Spearman’s R	0.522	—	—
	gl	37	—	—
	*p*	0.00066	—	—
NLI	Spearman’s R	−0.196	0.036	—
	gl	37	37	—
	*p*	0.231	0.830	—
CRP	Spearman’s R	−0.213	−0.243	0.358
	Gl	37	37	37
	*P*	0.193	0.136	0.025

Note: Phase angle (PhA), Spearman’s correlation coefficient (Spearman’s), degrees of freedom (gl), *p*-value (*p*), Neutrophil/Lymphocyte Index (NLI), C-reactive protein (CRP).

## Data Availability

The data supporting the findings of this study are available from the corresponding author upon reasonable request.

## Abbreviations

The following abbreviations are used in this manuscript:

TP	Total proteins
HCT	Hematocrit
NLI	Neutrophil/Lymphocyte Index
LPI	Lymphocyte–Platelet Index
CRP	C-reactive protein
PhA	Phase angle
gl	Degrees of freedom
CKD	Chronic kidney disease
CCI	Charlson Comorbidity Index
HD	Hemodialysis
